# Mitochondrial targeted catalase suppresses invasive breast cancer in mice

**DOI:** 10.1186/1471-2407-11-191

**Published:** 2011-05-23

**Authors:** Jorming Goh, Linda Enns, Soroosh Fatemie, Heather Hopkins, John Morton, Christina Pettan-Brewer, Warren Ladiges

**Affiliations:** 1 Department of Comparative Medicine University of Washington, Seattle WA 98195, USA; 2 Interdisciplinary Program in Nutritional Sciences, University of Washington, Seattle WA 98195, USA

## Abstract

**Background:**

Treatment of invasive breast cancer has an alarmingly high rate of failure because effective targets have not been identified. One potential target is mitochondrial generated reactive oxygen species (ROS) because ROS production has been associated with changes in substrate metabolism and lower concentration of anti-oxidant enzymes in tumor and stromal cells and increased metastatic potential.

**Methods:**

Transgenic mice expressing a human catalase gene (mCAT) were crossed with MMTV-PyMT transgenic mice that develop metastatic breast cancer. All mice (33 mCAT positive and 23 mCAT negative) were terminated at 110 days of age, when tumors were well advanced. Tumors were histologically assessed for invasiveness, proliferation and metastatic foci in the lungs. ROS levels and activation status of p38 MAPK were determined.

**Results:**

PyMT mice expressing mCAT had a 12.5 per cent incidence of high histological grade primary tumor invasiveness compared to a 62.5 per cent incidence in PyMT mice without mCAT. The histological grade correlated with incidence of metastasis with 56 per cent of PyMT mice positive for mCAT showing evidence of pulmonary metastasis compared to 85.4 per cent of PyMT mice negative for mCAT with pulmonary metastasis (p ≤ 0.05). PyMT tumor cells expressing mCAT had lower ROS levels and were more resistant to hydrogen peroxide-induced oxidative stress than wild type tumor cells, suggesting that mCAT has the potential of quenching intracellular ROS and subsequent invasive behavior. The metastatic tumor burden in PyMT mice expressing mCAT was 0.1 mm^2^/cm^2 ^of lung tissue compared with 1.3 mm^2^/cm^2 ^of lung tissue in PyMT mice expressing the wild type allele (p ≤ 0.01), indicating that mCAT could play a role in mitigating metastatic tumor progression at a distant organ site. Expression of mCAT in the lungs increased resistance to hydrogen peroxide-induced oxidative stress that was associated with decreased activation of p38MAPK suggesting ROS signaling is dependent on p38MAPK for at least some of its downstream effects.

**Conclusion:**

Targeting catalase within mitochondria of tumor cells and tumor stromal cells suppresses ROS-driven tumor progression and metastasis. Therefore, increasing the antioxidant capacity of the mitochondrial compartment could be a rational therapeutic approach for invasive breast cancer.

Please see related commentary article: http://www.biomedcentral.com/1741-7015/9/62

## Background

The chance of developing invasive breast cancer during a woman's lifetime is approximately 1 in 8 and more than 40,000 American women die of metastatic disease each year [1]. Despite making significant progress in elucidating the molecular mechanisms for breast cancer initiation and progression, effective treatments against metastatic progression remain elusive. Treatment of metastatic disease has an alarmingly high rate of failure because effective targets have not been identified. Inherent or acquired tumor drug resistance and dose-limiting toxicity limit many agents used in the treatment of breast cancer. This is observed through the lack of success in conventional chemotherapeutic trials, as well as the inability to prevent metastatic growth by surgical resection of the primary tumor.

It has recently been reported that mitochondrial polymorphisms associated with alterations in mitochondrial function play a role in women's risk for invasive breast cancer [2] suggesting a role for reactive oxygen species (ROS) production. In transformed epithelial cells, constitutively activated mitogenic pathways increase intracellular ROS, and activated metabolic pathways further increase the levels of endogenous ROS [3]. A decline in mitochondrial energy production can generate increased ROS, which cause mitochondrial mutations and additional ROS production [4,5]. As a consequence of mitochondrial dysfunction, cells are chronically subjected to a pro-oxidative environment [6], that is associated with tumor invasiveness [7], changes in tumor substrate metabolism [8], a lower concentration of anti-oxidant enzymes in tumor cells [9,10], and increased production or reduced clearance of ROS [11]. The molecular mechanisms of ROS-driven tumor progression are not well understood, but it is important to consider that sub-lethal concentrations of ROS are second messengers that up-regulate the expression of pathways involved in tumor growth and metastasis, such as p38MAPK [12]. Lethal concentrations of ROS may have the opposite effect and trigger cell death pathways for tumor cells [13].

ROS-driven pathways likely function in stromal cells as well as tumor cells. Recent work has shown that cancer associated fibroblasts undergo tumor mediated oxidative stress, which can then drive metabolic and mutagenic activities of tumor cells [14]. The suggestion is that stromal fibroblasts undergo aerobic glycolysis to generate energy-rich metabolites such as lactate and pyruvate, which are directly used by tumor cells to support oxidative phosphorylation. The resultant oxidative stress is significant to promote genomic instability in adjacent cancer cells, indicating that the tumor stroma can potentially increase cancer cell aggressive behavior via a bystander effect.

There is evidence to suggest that mitochondrial-generated oxidative stress can be attenuated with clinically relevant health benefits by increasing the concentration of mitochondrial antioxidant enzymes [15], or by ectopically expressing antioxidant enzymes within mitochondria [16,17]. Strategies to target cofactor-independent antioxidants, such as catalase, within the mitochondrial membrane would be predicted to effectively eliminate H_2_O_2 _at its source and prevent the formation of hydroxyl radicals and the subsequent cellular damage that can lead to a protumor environment. We report in this paper that transgenic expression of mitochondrial targeted catalase in a clinically relevant mouse model of invasive breast cancer decreases primary tumor invasiveness and metastatic tumor severity.

## Materials and methods

### Animals

FVB/N-Tg (MMTV-PyMT) 634 Mul/J transgenic males on a 100% FVB background [18] were obtained from Jackson Labs and crossed with mitochondrial targeted (mCAT) transgenic females on a congenic C57/BL6 background [17]. PyMT is a membrane bound polypeptide that can be regarded as an active analogue of a receptor that harbors docking sites for a number of effecter proteins used by tyrosine kinase receptors to stimulate mitogenesis. In fact, the same signaling pathways are activated as for ErbB2, an oncogene amplified or overexpressed in approximately 30 per cent of human breast cancers [19]. Mammary gland specific overexpression of PyMT using the MMTV promoter results in multifocal mammary adenocarcinomas with relatively short median latency and nearly 100 per cent metastasis to lungs and less frequently to lymph nodes [18]. The model shares numerous characteristics with human breast tumors. First, tumors develop with high penetrance and show gradual loss of estrogen and progesterone receptors. Second, the full multistage progression from hyperplasia to full-blown malignancy and metastasis is represented. Third, metastatic potential appears to be independent of hormonal fluctuations with a reproducible and measurable progression rate. A recent report has shown that PyMT transgenic mice on the C57BL/6 background have a longer mammary tumor latency (92 days) compared to PyMT on the FVB background (53 days) [20]. The incidence of lung metastasis is only slightly reduced. Our own data show that PyMT mice on the FVB background crossed with wild type C57BL/6 mice have a consistent and reproducible tumor latency time period of 70 days and still maintain a high incidence of lung metastasis (unpublished observations). Therefore, PyMT × mCAT F1 mice represent a useful and relevant model to study the effect of mCAT on tumor progression. Animals were kept in ventilated cages (4 to 5 per cage) in a specific pathogen free facility at the University of Washington. Mice were fed standard chow and provided reverse osmosis water. All supplies entering the facility were autoclaved. Rooms were kept at a 12-hour light/dark cycle, maintained at 70-74°F, 45-55% humidity with 28 changes per hour. Sentinel mice were tested every quarter and were negative for standard mouse pathogens.

Experimental F1 cohorts included PyMT × mCAT F1 transgenic (N = 18), PyMT transgenic × mCAT wild type (WT) (N = 12), PyMT WT × mCAT WT (N = 9), and PyMT WT × mCAT transgenic (N = 13) mice. The experimental procedure was repeated and included PyMT × mCAT (N = 15) and PyMT × WT (N = 11) littermates. Mice were palpated weekly for mammary tumors beginning at 60 days of age. Primary tumor incidence was determined by the presence of palpable mammary tumors for each genotype. Mice were sacrificed at 110 days of age by carbon dioxide asphyxiation, followed by necropsy. Standard necropsy procedures were performed. All palpable mammary tumors were excised, weighed and fixed with 10% neutral buffered formalin. Primary tumor volumes were determined by measuring in 3 dimensions with digital calipers and calculated as the product of length, width and height. Tissue samples were transferred into 70% ethanol after 24 hours. Fixed tissues were then paraffin embedded and 5 μM sections obtained for hematoxylin and eosin (H&E) staining. All experiments were approved by the University of Washington Institutional Animal Care and Use Committee.

### Primary cell cultures

Mammary tumors were aseptically excised from the fourth mammary fat pad from PyMT mice positive or negative for mCAT. Tumor explants were then washed with PBS and serum-free DMEM, placed in sterile petri dishes and mechanically dissociated and transferred to separate sterile petri dishes and DMEM with 20%FBS and 1% penicillin/streptomycin (Gibco/BRL Life Tech, Carlsbad, CA). Cells were then placed in an incubator at 37°C and 5% CO_2_. Media was replaced every 2-3 days until cells were near confluence. After confluence, cells were trypsinized and passaged onto multiple petri dishes and fed DMEM with 10% FBS and 1% penicillin/streptomycin. Once cells from the first passage became confluent, they were trypsinzed, passaged and frozen with freezing medium (DMEM with 20% FBS and 10% DMSO) and stored in liquid nitrogen. Lungs were harvested and treated in the same manner as the mammary tumors.

### Pathological assessment

H&E stained tumor tissues were assessed for morphology and general appearance, including: i) number of adenoma and carcinoma per tissue, ii) scirrhous response (scored from 0 to 4), iii) necrosis (scored from 0 to 4), iv) squamous metaplasia (scored from 0 to 2), v) inflammation (scored from 0 to 3), vi) degree of anaplasia (scored as either 0 or 1) and vii) invasion (scored from 0 to 3). Invasive foci were characterized by neoplastic acini or single cells present in the surrounding tissues including the mammary fat pad adipose tissue and deep underlying striated musculature. Invasion scores were applied with increasing severity as grade 1, a small single focus less than 20 cells or 1 acini; grade 2, multiple small foci consisting of single cells or small single acini; and grade 3, multiple invasive acini within multiple foci. Pulmonary metastatic foci and tumor burden were quantified from paraffin-embedded, H&E stained lung tissue sections. Slides were scanned to virtual digital files using Nanozoomer (Hamamatsu, Hamamatsu City, Japan). Images were magnified to 2.5× and were captured and used for quantification. The number of metastatic foci was counted in the entire 2.5× image. Metastatic incidence was defined as the number of mice bearing metastatic foci relative to the number of mice that did not have any metastatic foci. For metastatic tumor burden quantification, the ratio of tumor area to total lung surface area (square mm of pulmonary metastatic foci/square cm of lung tissue) was quantified using the Nikon Basic Research Image analysis software package.

### Ki-67 immunohistochemistry

Ki-67 is a cancer antigen that is found in rapidly growing, dividing cells but is absent in the rest phase of cell growth. To identify cells expressing ki-67, formalin-fixed mammary gland sections were immunostained using rabbit polyclonal to ki-67 proliferation marker (Abcam, Cambridge, MA Cat No. ab15580, Lot No. 465068, 0.5 mg/mL) in an automated immunstainer. Slides were deparaffinized for 30 minutes at 60°C. Antigen retrieval was achieved by adding Bond HIER 2 (EDTA) for 20 minutes, at 100°C. Endogeneous peroxidase activity was blocked using Leica Bond Peroxide block, 5 minutes at RT. To eliminate nonspecific interactions of secondary antibodies, tissues were incubated with 10% normal serum from the same host that had been used to generate the secondary antibodies. Sections were incubated for 20 minutes using diluted normal blocking serum, serum excess removed and primary antibody, Rabbit Ki-67 1:2000 (0.25 mg/mL), or Rabbit isotype 1:5000 (0.2 mg/mL) as negative control, were added to the slides after diluted in Leica primary antibody diluent for 15 min at RT. Specific reactivity was detected using Leica Bond Polymer DAB Refine, 8 minutes at RT and Leica Bond Mixed Refine (DAB) detection 10 minutes at RT. Tissues were counterstained for 10 seconds in Harris Hematoxylin followed by two rinses in H_2_O and cleared with Xylene. Slides were mounted with a cover slip. The number of ki-67 labeled cells was determined by counting positive cells in a grid of eight squares encompassing the entire plane of view at 200× magnification per slide. The ki-67 labeling index was calculated as the average per cent positive cells.

### Catalase assay

Cells were grown to confluence in DMEM supplemented with 10% FBS and placed in a two-chambered slide system. Cells were then washed with PBS, and fixed with 4% paraformaldehyde, permeabilized for 30 minutes with TBST (50 mM Tris-Hcl, pH 8.0, 140 mM Nacl, 2.7 mM KCl, 0.1% Triton-X100 and 0.3% BSA) at room temperature, and stained with primary anti-human catalase (sc43282, Santa Cruz), followed by Alexa-488 (Invitrogen) secondary antibodies, and counterstained with Hoechst 33342. Images were obtained using a Zeiss-confocal microscope.

### ROS quantitation

Cells were grown to confluence in DMEM supplemented with 10% FBS, at 37°C and 5% CO2. Confluent cells were passaged and a cell-count was performed. 10,000 cells were seeded per well in a 96-well plate. Cells were then incubated for 4 hours at 37°C and 5% CO_2_, to allow adhesion. Cells were then probed for ROS using a DCFH-DA-based, intracellular ROS assay (STA-342; Cell Biolabs, CA), as per manufacturer's instructions. In brief, media was removed and cells were washed twice with PBS (14190-250; Gibco/BRL Life Tech, Carlsbad, CA). 100 μl of the cell-permeable, fluorogenic probe 2'-7'-dichlorodihydrofluorescein diacetate (DCFH-DA), diluted in serum-free DMEM, was added to each well and cells were incubated for 30 minutes. Wells were washed again, thrice, with PBS, at which point the cells were lysed and fluorescence was quantified with a microplate reader (Perkin Elmer Victor3 V multi-mode)set at 485 nm (excitation) and 535 nm (emission). A corresponding plate, also seeded at 10,000 cells per well and pre-incubated for 4 hours under the same conditions, was assayed for rate of mitochondrial electron transport using the tetrazolium salt WST-1 as per manufacture instructions (1-644-807; Roche, Mannheim, Germany). In brief, cells were incubated for 1.5 h in 100 μl of serum-free DMEM with 10 μl of WST-1 reagent, and then absorbance at 450 nm, which is directly related to the rate of activity of mitochondrial dehyrogenase, was read using a microplate reader. The amount of ROS in each well was standardized to the rate of mitochondrial electron transport, and the probability of significant difference was calculated using a Student's t-test.

### Cell viability

Cells were plated in DMEM with 10% FBS and 1% Pen-Strep (Gibco/BRL Life Tech, Carlsbad, CA) in 96-well plates. After 24 hours, media was removed, and cells were treated with 0, 0.5, 0.7, 0.85, 1.0 and 2.0 mM H_2_0_2 _(H324-500; Fisher Scientific, Auburn, WA), in DMEM/10% FBS/1% Pen-Strep. After being incubated with their respective treatments for 4 h at 37°C in a humidified atmosphere with 5% CO_2_, all wells were replaced with 100 μl of fresh media/10% FBS/1% Pen-Strep. Cells were allowed to recover for another 48 h at 37°C in a humidified atmosphere with 5% CO_2_. 10 μl of Cell Proliferation Reagent WST-1 (BD Biosciences, Mountain View, CA) was added to each well and cells were put back in the incubator for another 1.5 hours. Plates were then shaken thoroughly on a shaker, and the absorbance of each of the wells was measured using a microplate reader at 450 nm. Wells without cells were used as a blank, and average absorbances for each H_2_O_2 _treatment were standardized relative to the absorbance at 0 mM H_2_O_2_. Viability was recorded as per cent viable cells.

### Western immunoblotting

Cells were treated with 0, 100 and 500 μM H_2_O_2 _(H324-500; Fisher Scientific, Auburn, WA) for 4 hours. They were then washed with PBS (14190-250; Gibco/BRL Life Tech, Carlsbad, CA), trypsinized for 5 to 10 min at 37°C, and pelleted. Cells of each plate were resuspended in 200 μl RIPA buffer (89901; Thermo Scientific) with 10 μl each of protease and phosphatase inhibitors P8340, P2850 and P5726 (Sigma-Aldrich) and then sonicated to extract total cytosolic protein. Westerns were run with 20 ug protein per lane. Primary antibodies were used to target the following: Phospho-p38MAPK (Thr180/Try182) and p38MAPK (#9211 and #9212; Cell Signaling Technology). Primary antibodies were then labeled with an HRP-conjugated secondary antibody (ab6721; Abcam). HRP was detected by using an ECL Western Blotting Analysis System (RPN2109; Amersham Biosciences). A densitometric analysis was done to quantitate individual intensities of the bands.

### Statistical analysis

Differences in the proportions of primary and metastatic tumor incidence as well as proportion of high-grade primary lesions between PyMT expressing mCAT and PyMT negative for mCAT were evaluated using a 2 × 2 contingency table. A Fisher's exact test was used to test for statistical differences, with significance set at p ≤ 0.05. Differences in mean tumor volumes, tumor weights/burden, as well as relative fluorescent units and relative absorbance between the two genotypes were carried out using student's t-test with significance set at p ≤ 0.05.

## Results

### Mitochondrial catalase decreases invasiveness of primary breast tumors

**Figure 1:**
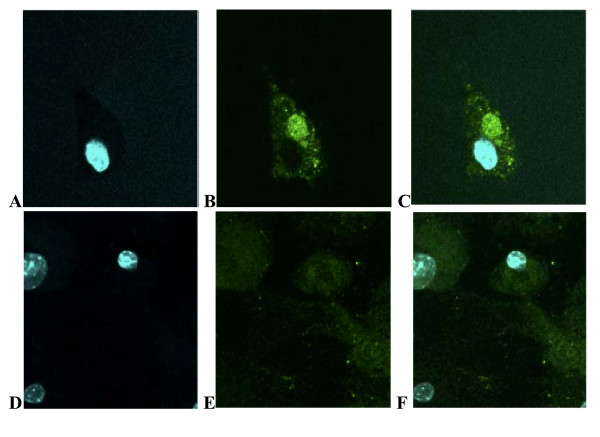
**Catalase is targeted to mitochondria of tumor cells from mCAT positive PyMT mice. **Mammary tumor cells stained with human primary anti-catalase and Alexa 488 secondary antibodies and counter-stained with Hoechst 33342 staining for the nucleus. **A-C **showing nuclear stain, mitochondrial catalase stain and overlap in PyMT cells expressing mCAT and **D-F **in PyMT cells negative for mCAT, respectively.

**Figure 2:**
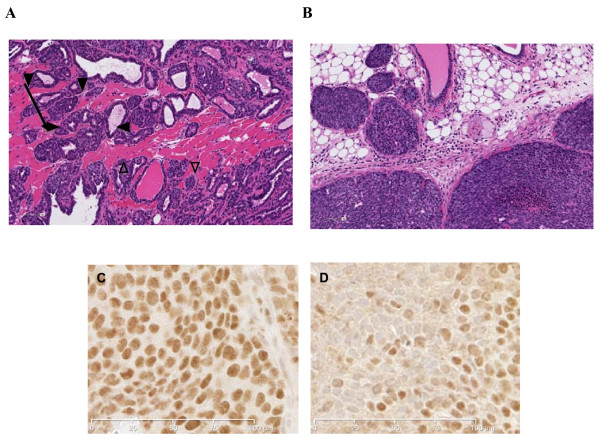
**Histological sections of mammary tumors stained with H and E or ki-67 antibody**. **A**. Invasion was identified based on growth patterns such as glandular infiltration into surrounding skeletal muscle fibers (arrow) in tumors from PyMT mice negative for mCAT. Invasive foci were scored from 0 (none) to 3 (multiple invasive fronts). **B**. Low grade, encapsulated adenomas (closed arrow head) were more common in PyMT mice expressing mCAT. **C**. Ki-67 staining of PyMT primary tumor cells negative for mCAT showed a labeling percent of 56. **D**. Ki-67 staining of PyMT primary tumor cells positive for mCAT showed a labeling percent of 21. The difference in ki-67 labeling between mCAT positive and mCAT negative PyMT primary tumor cells was significant at p ≤ 0.05.

The presence of mitochondrial catalase (Figure 1) had a profound affect on the invasive phenotype of the primary tumor, with an average invasive grade of 1.8 ± 0.5 while the average invasive grade of tumors in the absence of mCAT was 2.8 ± 0.5, p ≤ 0.03 (Table 1). Only 13 per cent of tumors with mCAT displayed a histologically invasive phenotype of grade 3, while 63 per cent of tumors without mCAT had a histologically invasive grade of 3. Invasiveness was represented by glandular infiltration into surrounding skeletal muscle fibers (Figure 2A). In contrast, a greater number of mammary tumors from PyMT mice positive for mCAT showed features of well-encapsulated adenoma with peritumoral inflammation (Figure 2B). The difference of high-grade invasiveness in primary tumors was reflected in ki-67 labeling with 56 per cent positive in PyMT cells (Figure 2C) compared to 21 per cent positive in PyMT cells expressing mCAT, p ≤ 0.05 (Figure 2D). The difference in high-grade invasiveness in primary tumors was also reflected in metastatic tumor incidence to the lungs. Pulmonary metastasis was seen in 85 per cent of PyMT mice expressing the wild type mCAT allele, while only 56 per cent of PyMT mice expressing mCAT showed pulmonary metastasis (P ≤ 0.05) (Table 1). Although the histological phenotype of the primary tumors was drastically different between PyMT mice positive or negative for mCAT, primary tumor burden was not statistically different (Table 1), suggesting that mCAT may be reducing stromal infiltration and cellular motility and migration of primary tumors, either through direct effects on the tumors themselves, or *via *some cross-talk with stromal cells.

**Table 1:** Breast tumor invasiveness in polyoma middle T (PyMT) transgenic mice positive or negative for mitochondrial targeted catalase (mCAT).

PyMT Genotype	Number of mice	Primary tumor incidence	Average Invasion Grade	Metastatic tumor incidence	Metastatic tumor ** burden**^**a**^
WT	23	100%	2.8 ± 0.5	85.4%	1.3 ± 0.3
mCAT	32	97%	1.8 ± 0.5*^b^	56.0%**	0.1 ± 0.02***

a) Metastatic tumor burden expressed as mm^2 ^tumor/cm^2 ^lung. b) Significant at P ≤ 0.03*; P ≤ 0.05**, or P ≤ 0.01***.

Mice were from two separate experiments that were each terminated at 110 days of age.

### Primary tumor cells from mCAT Tg mice are resistant to oxidative stress

**Figure 3:**
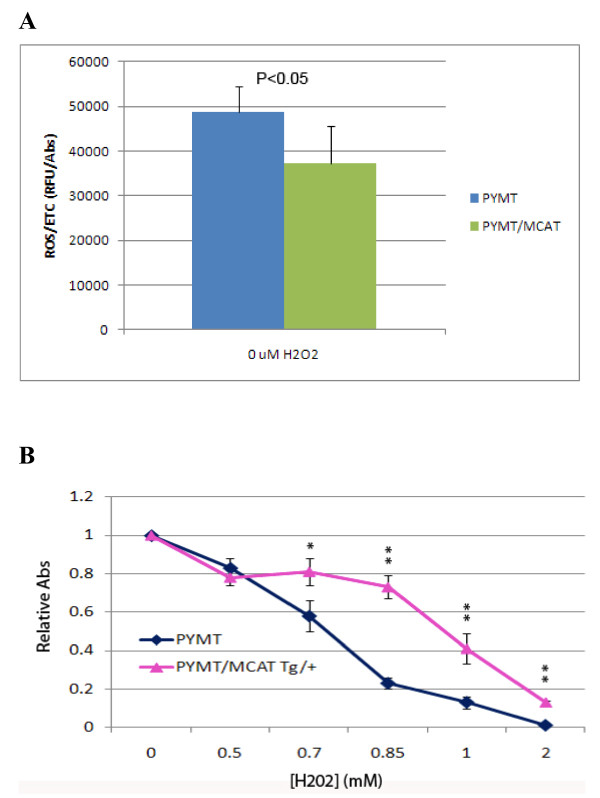
**Tumor cells expressing mCAT are resistant to oxidative stress.** A. Primary PyMT tumor cells expressing mCAT have lower levels of ROS compared to tumors cells from wild type littermates. p ≤ 0.05. **B**. PyMT mammary tumors expressing mCAT are resistant to H2O2 compared to PyMT mammary tumors negative for mCAT, *p < 0.05, **p < 0.001.

In order to provide preliminary insight into the role of ROS signaling in the invasive phenotype, we determined whether mammary tumor cells expressing mCAT were more resistant to oxidative stress than mammary tumor cells from WT littermates, and looked to see if they had lower intracellular concentrations of ROS. PyMT mammary tumor cells positive for mCAT showed reduced ROS concentrations compared with PyMT tumor cells negative for mCAT (Figure 3A), and were more resistant to the toxic affects of increasing concentrations of H_2_O_2 _(Figure 3B). Therefore, the presence of mCAT in PyMT cells appears to quench intracellular ROS making them less sensitive to the invasive promoting effects of ROS, and suggesting that targeting mitochondria within primary tumor cells with catalase is a potentially relevant clinical strategy to prevent, or at least slow the rate, of invasive breast cancer progression.

### Metastatic tumor burden is decreased in the presence of mCAT

**Figure 4:**
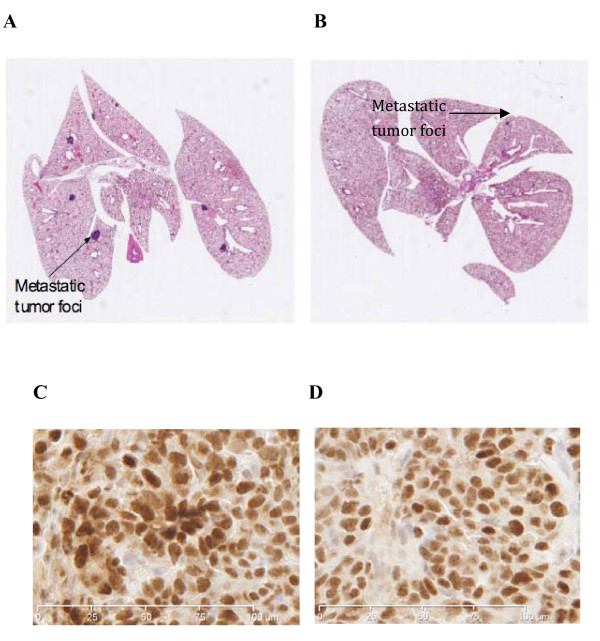
**Metastatic tumor burden and proliferative capacity are decreased in the presence of mCAT**.  Surface area of metastatic foci in lungs of PyMT/WT mice (A) was significantly larger than metastatic foci in lungs of PyMT/mCAT mice (B). Arrows indicate metastatic tumors. Original magnification was at 2.5×. **C**. Ki-67 staining of PyMT metastatic tumor cells negative for mCAT showed a labeling percent of 23. **D**. Ki-67 staining of PyMT metastatic tumor cells positive for mCAT showed a labeling per cent of 6. The difference in ki-67 labeling between mCAT positive and mCAT negative PyMT metastatic tumor cells was significant at p ≤ 0.01.

PyMT mice positive for mCAT showed a more than two-fold decrease in the average number of metastatic foci in the lungs, as well as a 10-fold decrease in the metastatic tumor burden, compared with mCAT negative PyMT littermates (0.1 square mm per square cm of lung tissue versus 1.3 square mm per square cm of lung tissue, respectively, P ≤ 0.01) (Table 1). Figure 4 shows a representative scanned H&E stained slide of lungs from an mCAT negative (4A) or mCAT positive (4B) PyMT mouse, where larger metastatic tumor foci are seen in the absence of mCAT compared to much smaller foci in the presence of mCAT. The difference was reflected in ki-67 labeling with 23 per cent positive in PyMT cells (Figure 4C) compared to 6 per cent positive in PyMT cells expressing mCAT, p ≤ 0.01 (Figure 4D). Therefore, mCAT appears to play a role in suppressing progression and proliferation of tumor foci at a distant site of metastasis.

### Expression of mCAT in the lungs increases ROS resistance

**Figure 5:**
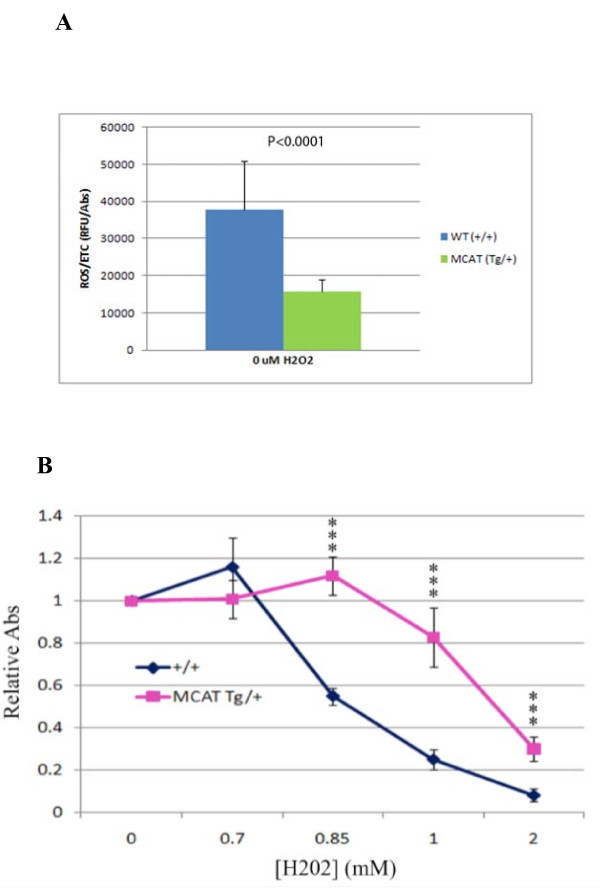
**Resistance to ROS is increased in lung fibroblasts expressing mCAT**.  A) Lung fibroblast cells expressing mCAT have lower levels of ROS compared to lung fibroblast cells from wild type littermates, p ≤ 0.05, and B) are more resistant to H2O2 challenge, ***p < 0.001.

We asked the question whether pulmonary stromal cells that expressed mCAT displayed any resistance to oxidative stress. Lung fibroblasts were obtained from mice that were either wild-type or transgenic for mCAT. ROS levels were decreased in lung fibroblasts from mCAT positive mice compared to wt mice (Figure 5A). When treated with H_2_O_2 _and their viability compared, lung fibroblasts that expressed mCAT were more resistant to H_2_O_2 _than wild-type lung fibroblasts and displayed greater viability at the same concentration of H_2_O_2 _(Figure 5B), suggesting that mCAT is capable of neutralizing the effect of ROS in cells in the pulmonary microenvironment.

### ROS are associated with activation of p38MAPK

**Figure 6:**
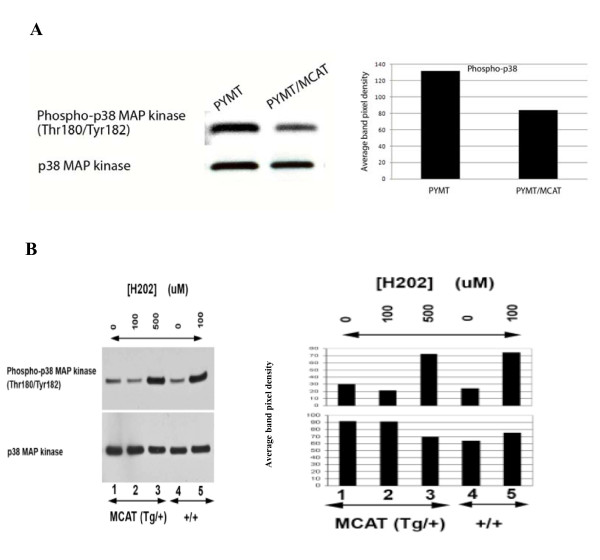
**Phosphorylation of p38MAPK is decreased in the presence of mCAT. A.** Immunoblot of total protein extract from MCAT (Tg/+) and WT (+/+) primary tumor cells. **B**. Immunoblot of total protein extract from MCAT (Tg/+) and WT (+/+) lung fibroblasts, treated for 4 hours with 0, 100 and 500 μMH_2_. Lung fibroblast cells expressing mCAT attenuate phosphorylation of p38MAP kinase in the presence of increased levels of oxidative stress. ***p < 0.0001.

One of the molecular mechanisms helping mediate the ROS resistant process may be the p38MAPK pathway [12]. We show that increasing levels of ROS are associated with increases in phosphorylation of p38MAPK, but this phosphorylation is dramatically attenuated in the presence of mCAT (Figure 6A), suggesting that p38MAPK could be part of a downstream ROS signaling pathway.

## Discussion

Our results show that PyMT transgenic mice expressing mCAT have reduced primary tumor invasiveness and decreased pulmonary metastatic tumor incidence and burden, compared with PyMT mice expressing the WT allele. These findings are in agreement with our previously reported observations that old transgenic mice expressing mCAT had less invasive epithelial cancers, compared with wild type littermates [17]. A possible explanation for the protective effect of mCAT in the PyMT model system could be due to an abrogation of ROS-dependent signaling required for attaining invasive behavior. Our data show that PyMT mammary tumors expressing mCAT are mainly comprised of well-encapsulated adenomas (Figure 2B), whereas PyMT mammary tumors negative for mCAT had increased invasion represented by glandular infiltration into skeletal muscle (Figure 2A). It is possible that mCAT alters ROS-dependent signaling involved in epithelial-to-mesenchymal transition (EMT), a process associated with metastasis. Hypoxia, which is common in tumors [21], stimulates the production of mitochondrial generated ROS [22], which can promote EMT and metastasis through activation of a number of different factors [23]. Increases in ROS that have no effect on normal cells can actually promote cancer cell growth [24,25], and we have shown that mCAT confers resistance of PyMT cells to increasing levels of H_2_O_2_. Hypoxia in peri-necrotic areas surrounding a tumor may release vascular endothelial factors, which act as powerful chemoattractants for tumor-associated macrophages (TAMs) in murine models of tumorigenesis [26]. These TAMs support tumor progression [27] by promoting invasion, angiogenesis and metastasis [28] as a consequence of tissue hypoxia and the subsequent generation of ROS. It has also been reported that endothelial cells and fibroblasts along with TAMs up-regulate monocyte chemoattractant protein (MCP)-1 [29]. Thus, a complex cross-talk between ROS, stromal cells and tumor cells occurs to promote invasive behavior.

An additional explanation for the protective effect of mCAT in the PyMT model system is based on the concept that high levels of ROS produced by cancer cells increase their motility by degrading the surrounding extracellular matrix, leading to increased potential for migration and invasion. It is believed that metastasis following surgical removal of tumors is in part due to ROS generated by the procedure, an idea that is supported by the fact that superoxide dismutase (SOD) derivatives inhibit the growth of micrometastasis after surgical removal of tumors in mice [30]. Mitochondrial DNA (mtDNA) is also known to play a role in the metastatic behaviour of tumor cells. If mtDNA in a poorly metastatic tumor cell line is replaced with mtDNA from a highly metastatic cell line and vice versa, recipient tumor cells acquire the metastatic potential of the mtDNA donor. Mutations in mtDNA occur at high frequency in tumor cells, and those that cause overproduction of ROS are associated with increased metastatic potential. Pretreatment of these cells with ROS scavengers can decrease their ability to metastasize [31]. ROS generated by H_2_O_2 _at sub-lethal concentrations can act as an intracellular second messenger to increase transcription of various genes that enhance proliferation of tumor cells in metastatic colonies [32]. Finally, disrupting the mitochondrial respiratory chain leads to increased ROS and has been shown to increase the motility and invasiveness of breast cancer cells through mechanisms mediated by cytokines such as CXCL14 [6].

Other mechanisms may also be involved in the protective affect of mCAT. A recent study showed that cytokeratin positive tumor cells were found in bone marrow of PyMT mice as early as 4 to 6 weeks of age, coinciding with atypical ductal hyperplasia (ADH) and ductal carcinoma *in situ *(DCIS) in mammary tissue [33]. This suggests that disseminated tumor cells from the mammary tissue may be present at distant organs at an early stage of tumor progression and remain dormant. The fact that reduced metastatic foci were observed in PyMT lungs positive for mCAT suggests that mCAT could be either reducing primary tumor dissemination at an early stage of tumorigenesis, or attenuating growth signals at the secondary site, or both. A putative mechanism for this could involve the cellular stress sensor p38MAPK, where its expression is positively correlated with invasive breast cancers [34], as well as EMT and cell migration [35]. Our immunoblots show that in mCAT transgenic lung fibroblasts, p38MAPK was phosphorylated at a higher concentration of H_2_O_2_, compared with wild-type lung fibroblasts, where p38MAPK was phosphorylated at a 5-fold lower concentration. Our interpretation is that PyMT lung fibroblasts positive for mCAT would have a lower expression of p38MAPK, which could be attributed to decreased cellular stress, presumably in this context, lower oxidative stress. A reduced expression of p38MAPK in the lung fibroblasts may translate to lower E- and P-selectin expression, thus attenuating the metastatic potential of tumor cells by weaker attachment and seeding at the target organ [36]. Increased anti-oxidative capacity of surrounding stromal cells in the PyMT lungs positive for mCAT may thus alter their molecular cross-talk with metastatic tumor cells and hence inhibit their colonization [37].

Recent data have suggested an association with ROS levels in tumor epithelial cells and stromal fibroblasts [14]. Signals from tumor epithelial cells initiated metabolic dysfunction resulting in increased ROS that directly affected tumor cells by enhancing their invasive and metastatic qualities. That increased levels of ROS enhance tumorigenesis may seem a paradox in view of recent findings that increased ROS can also be associated with increased apoptosis of tumor cells and decreased tumor proliferation [13]. However, it may be that it is a matter of ROS concentration, ie., high concentrations of ROS generated in overwhelming and severe mitochondrial damage trigger tumor cell apoptosis as a feature of overzealous tumor cells. On the other hand, tumor cells that have adapted more to a controlled environment are able to use ROS to their advantage by inducing stromal fibroblasts, as well as other stromal cells, as mediators of survival.

## Conclusions

Our findings describe how transgenic expression of mCAT in PyMT mammary cancer decreases primary tumor invasiveness and suppresses pulmonary metastases. From a clinical perspective, mitochondrial targeted catalase should be considered as a potential adjuvant treatment strategy for further investigation to help prevent metastasis in women diagnosed with invasive breast cancer.

## Abbreviations

mCAT: mitochondrial targeted catalase; ROS: reactive oxygen species; PyMT: polyoma middle T oncoprotein; MMTV: mouse mammary tumor virus; p38 MAPK: mitogen activated protein kinase; akt: protein kinase b; EMT: epithelial-to-mesenchymal transition; TAM: tumor-associated macrophages; MCP-1: monocyte chemoattractant protein-1; SOD: superoxide dismutase; mtDNA: mitochondrial DNA; CXCL14; chemokine (C-X-C motif) ligand 14; ADH: atypical ductal hyperplasia; DCIS: ductal carcinoma *in situ*.

## Competing interests

The authors declare that they have no competing interests.

## Authors' contributions

WL conceived and supervised the project, analyzed and interpreted the results and co-drafted the manuscript. JG co-drafted the manuscript, performed primary cell culture experiments, ROS assays, animal dissections, tumor measurements and performed statistical analyses, and analyzed and interpreted the results. LE contributed to the discussion of the manuscript, performed western immunoblot, ROS and cell viability assays. SF performed fluorescent microscopy experiments. HH and CPB participated in animal dissections and tumor measurements. CPB performed Ki-67 immunohistochemistry assays. JM performed cell viability assays and primary cell cultures. All authors read and approved the manuscript.

## Pre-publication history

The pre-publication history for this paper can be accessed here:

http://www.biomedcentral.com/1471-2407/11/191/prepub
